# Tooth Decay in Alcohol Abusers Compared to Alcohol and Drug Abusers

**DOI:** 10.1155/2010/786503

**Published:** 2010-04-01

**Authors:** Ananda P. Dasanayake, Saman Warnakulasuriya, Colin K. Harris, Derek J. Cooper, Timothy J. Peters, Stanley Gelbier

**Affiliations:** ^1^Department of Epidemiology and Health Promotion, New York University College of Dentistry, 250 Park Avenue South—6th Floor, New York, NY 10003-1402, USA; ^2^Department of Oral Medicine, King's College London, Denmark Hill Campus, London SE5 9RW, UK; ^3^Department of Clinical Biochemistry, King's College London, Denmark Hill Campus, London SE5 9RW, UK; ^4^The Welcome Trust Centre for the History of Medicine at UCL, 210 Euston Road, London NW1 2BE, UK

## Abstract

Alcohol and drug abuse are detrimental to general and oral health. Though we know the effects of these harmful habits on oral mucosa, their independent and combined effect on the dental caries experience is unknown and worthy of investigation. We compared 363 “alcohol only” abusers to 300 “alcohol and drug” abusers to test the hypothesis that various components of their dental caries experience are significantly different due to plausible sociobiological explanations. After controlling for the potential confounders, we observe that the “alcohol and drug” group had a 38% higher risk of having decayed teeth compared to the “alcohol only” group (*P* < .05). As expected, those who belonged to a higher social class (OR = 1.98; 95%  CI = 1.43–2.75) and drank wine (OR = 1.85; 95%  CI = 1.16–2.96) had a higher risk of having more filled teeth. We conclude that the risk of tooth decay among “alcohol only” abusers is significantly lower compared to “alcohol and drug” abusers.

## 1. Introduction

Alcohol and drug dependence are conditions characterized by psychological, physiological, and pathological changes, all of which are directly relevant to dentistry [1]. The psychological effects and the personality changes in the abuser may affect the patient/dentist relationship as they take a reduced interest in seeking and paying for dental care. The physiological effect of alcohol intoxication may lead to the inability to understand and accept advice given by health care workers that may result in noncompliance. Pathological aspects of alcohol and drug abuse on dental and oral tissues have not been examined in detail except for its effects on the oral mucosa [2]. 

 We hypothesize that “alcohol only” abusers have a significantly different caries experience compared to “alcohol and drug” abusers due to a variety of biological reasons. We propose the following biological model to explain the potential association between alcohol and drug abuse and dental caries. Microbial oxidation of ethanol in saliva in alcohol abusers will result in the formation of acetaldehyde [3] that may further alter the cariogenic oral flora by reducing their levels [4]. Warnakulasuriya et al. have shown that certain alcoholic beverages in the UK contain high levels of fluoride and those who consume three cans of beer a day in the UK would receive the recommended daily upper limit of fluoride through beer alone [5]. As most alcoholics may consume more than three cans, their exposure to higher levels of fluoride via alcoholic beverages may reduce their caries susceptibility. Alcoholic beverages may also enhance the fluoride release in restorative materials such as compomers [6]. On the other hand, alcohol and drug abusers might experience dry mouth at night [7] and neglect both personal and professional oral health care [8]. They may also consume higher levels of refined carbohydrates [9] to satisfy their “munchies.” All of these might increase their risk of caries. 

**Figure 1:**
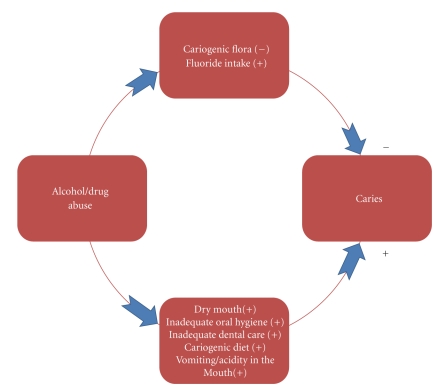
Hypothetical biological model to explain the association between alcohol and drug abuse and dental caries.

 However, it is unclear how the alcohol and drug abuse may affect different components of their overall caries experience. Figure 1 explains the scenario described above as an attempt to provide the basis for our hypothesis. It is important to evaluate the effect of these exposures independently, and in combination, to better understand the association between alcohol and drug abuse and different components of the dental caries experience.

Globally, reliable epidemiological data on dental caries of alcohol and drug abusers are scarce. The objective of this study, therefore, was to test the effect of “alcohol only” abuse and “alcohol and drug” abuse on selected components of the caries experience in abusers who are residents in South London. It would have been ideal to have another similar-sized comparison group of those who abuse “drugs only,” but we only had a limited sample of that group, and therefore, we will only describe the findings from that group as an adjunct to the main discussion.

## 2. Materials and Methods

The study group comprised of persons who attended the following clinical care facilities in south London between 1994 and 1999: A weekly out-patients' alcohol intervention clinic at King's College Hospital, the Drink Crises Centre (Voluntary Sector Residential Centre), Detoxification Units at The Maudsley and the Royal Bethlem Hospitals, the Community Drink/Drug Project Unit, a Rehabilitation Centre at St. Luke's Mission, and several local half-way housing units for chronic alcoholics. The study protocol was approved by the Research Ethics Committee of the King's Healthcare NHS Trust. Each volunteer was given an information sheet and a verbal explanation before being asked for written consent to participate in the study. All clinical care facilities were visited by one author (C.K.Harris) monthly/bimonthly subject to their availability. Using a feasibility sampling scheme all newly admitted subjects in residence or in attendance on the day of the visit were approached and invited to a dental and oral examination, except in situations where a Nurse Manager thought the person was too ill or would be unfit for an interview.

 A questionnaire was used to record the type of alcohol beverage used, its frequency and duration of use, smoking habits, and standard demographic data (see the appendix in the Supplementary Material available online at doi:10.1155/2010/786503). Any drug abuse, its duration and the type of drug used were also recorded. Any prescribed or self-administered medication for the patient was also recorded. The examiner administered the questionnaire to each subject at the interview. The questionnaire was pilot tested [10] using 107 subjects drawn from three of the centers listed earlier. The subjects included in the pilot study were not included in the present analyses. We had no means of testing the validity of self-reported data but our experience is that UK study subjects are less likely to under-report even harmful habits. 

 Standard demographic data including ethnicity were recorded. Patients were classified according to the Registrar General's socioeconomic classification [11, 12]. A comprehensive clinical oral examination was performed on each subject. The standard World Health Organization protocol for dental caries examination and categorization was used [13]. No radiographs were taken. Oral examination lasted approximately fifteen minutes and the questionnaire administration took about 30 minutes on average. Every attempt was made to mask the interviewer to the examination data and vice-versa. Detail examination methods are given elsewhere [10]. 

 Any abnormal findings and treatments required were reported to the patient on examination, the head of the unit at the institution where the patient was seen, and to the patient's General Medical Practitioner (GP), and the General Dental Practitioner (GDP). Patients not registered with a GDP were referred to the Primary Care Unit of the King's Dental School or to the St. Giles Trust for the Homeless in Camberwell, South London. 

 Data collected were managed and analyzed using SPSS Version 16. Univariate comparisons between the two groups were made using the independent samples *t*-test for quantitative variables and the chi-square test for categorical variables. Binary logistic regression models were developed for exploring both univariate and multivariate relationships; included in the latter were all variables with significance of 0.1 or less in the univariate analysis. Two-sided Type I Error probability ≤.05 was used as the level of significance. 

## 3. Results

There were 388 subjects who identified themselves as “alcohol only” abusers and 305 subjects who admitted to abusing both “alcohol and drugs.” We decided to exclude those who were edentulous. When the edentulous subjects were excluded from both groups, there were 363 “alcohol only” abusers and 300 “alcohol and drugs” abusers. Subjects were on average in their 3rd and 4th decades of life and predominantly White (over 90%) and male (over 75%). The “alcohol only” group was significantly older (43.5 ± 8.8 versus 35.4 ± 7.3 years; *P* < .001) and had abused alcohol for a longer period (22.9 ± 10.3 versus 16.6 ± 8.5 years; *P* < .001). However, their self-reported current smoking was significantly lower (84%) compared to the “alcohol and drugs” group (95%; *P* < .001; Table 1). There was no significant difference in mean weekly alcohol consumption (units per week) between the two groups (*P* = .60). 

**Figure 2:**
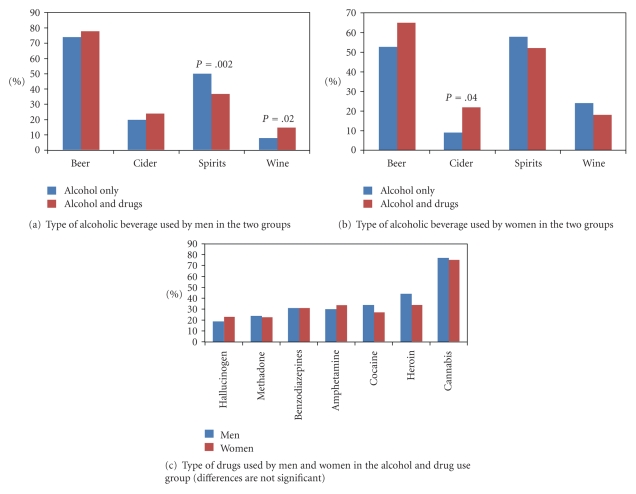
Alcohol and drug type used (%) by men and women in each group.

Types of alcohol and drugs used by men and women in each group are given in Figure 2. Significantly higher proportion of men in “alcohol only” group drank spirits and a lower proportion drank wine compared to the “alcohol and drugs” group (Figure 2(a)). Contrastingly, significantly lower proportion of women in the “alcohol only” group drank less cider (Figure 2(b)). Gender differences in the types of drugs used within “alcohol and drugs” group were not significant (Figure 2(c)).

The dental status and the caries experience are shown in Table 2. The “alcohol only” group had significantly fewer teeth, more missing teeth, and a higher DMFT value. Their D and F components however, were lower compared to the “alcohol and drug” group (though the F component failed to achieve statistical significance). 

In order to test if the lower D and F values in the “alcohol only” group are confounded due to other variables, we performed multivariate binary logistic regression analysis. Variables included in the multivariate model were the ones that were significant at the 10% level in the bivariate analysis (Table 3). We dichotomized the D component using the median of 0 versus 1+ and the F component using less than or equal to the median of 8 versus >8 to define the respective outcome variables for the D and the F components. Table 3 shows the variables that were associated with either the higher D or the higher F component of the caries experience. White race, “Alcohol and drug” abuse, and the amount of alcohol consumed per week were positively associated with a higher D component (at 10% level of significance). Higher social class and wine drinking were positively associated with a higher F component. Male gender and beer drinking reduced the risk of having a higher F component.

In the final multivariate model (Table 3), Whites (OR = 2.26; 95% CI = 1.15–4.45; *P* = .018) and “alcohol and drug” abusers (OR = 1.38; 95% CI = 1.01–1.89; *P* = .049) had a significantly higher D component. Those who belonged to a higher social class and drank wine had a significantly higher risk of having more filled teeth (*P* < .05). Beer drinkers had a lower risk (OR = 0.83) as we hypothesized, but the difference between beer drinkers and nonbeer drinkers was not statistically significant (95% CI = 0.58–1.19; *P* = .31). 

## 4. Discussion

Using over 600 alcohol and drug abusers, we observed that their total DMFT is around 16–18. However, the D component of the caries experience among alcoholics was significantly lower compared to those who abused both alcohol and drugs. Our multivariate analysis also confirmed that the alcohol and drug abusers in south London had a higher risk of having decayed teeth compared to “alcohol only” group. 

 Alcoholics and substance abusers are known to have poor oral health in other populations. In a survey of hospitalized alcoholic patients in Wyoming, USA, alcoholics had a three times higher permanent tooth loss than the national average for corresponding ages [14]. A smaller group of alcoholics in Maryland also had a higher number of missing teeth [15]. In a case-control study of 85 volunteer Finnish alcoholics, there were significantly fewer teeth and more remaining teeth with caries [16].

 Among drug abusers, higher rates of caries have been reported in Australia [17], Poland [18], Sweden [19], Holland [20], and Denmark [21]. Methadone users are also known to have a higher caries experience [22], which is now known as “meth mouth”.

 Before we interpret our findings, we need to examine the strengths and limitations of our study. This study is unique as we had over 600 predominantly adult White males included in the study from south London, minimizing the heterogeneity of the findings. However, among the study limitations are our feasibility sampling due to logistics, potential under or over reporting of self-reported data, and the inherent limitations in the field dental examinations. We however would argue that these limitations were randomly distributed (i.e., non-differentially) among both “alcohol only” and “alcohol and drug” abuse groups, thus biasing our estimates towards the null value. 

 It is not “earth-shattering” to state that alcohol and drug abusers have poor oral health. That was not the intention of this study. We wanted to further evaluate the effect of alcohol and drug abuse either alone or in combination on various components of the dental caries experience. As noted, our “alcohol only” group had fewer teeth and a higher DMFT. Alcohol is currently considered an independent risk factor for periodontal disease [23], and therefore, one can expect fewer teeth among alcohol users. Our “alcohol and drug” abuse group was significantly younger though they too consumed alcohol, but the significant age difference might also explain why the “alcohol only” group had significantly fewer teeth. What is interesting is that the amount of alcohol (units per week) consumed by subjects in each group was very high (over 280 units per week), but that was not statistically significantly different. This challenges our hypothesis that alcohol consumption reduces the decayed and filled component of DMFT. One possible explanation for this is that the potential higher consumption of refined carbohydrates by the “alcohol and drug” abuse group can override the “caries reducing” effect of “alcohol alone”. To support this notion, we looked at data from 76 subjects who only abused drugs without alcohol (as a part of the dissertation of one of the authors-C.K.Harris). These subjects came from the same clinics that gave rise to the study subjects included in this. Harris [24] reported that “drugs only” group had significantly higher decayed teeth (mean = 3.0; SD = 4.4) compared to the other two groups (*P* < .05) that are reported in this—“alcohol only” group = 0.95 (1.7) and “alcohol and drugs” group = 1.3 (2.5). 

As we argued in the introduction, it is possible that the “alcohol only” group has fewer decayed teeth due to fluoride in alcohol and/or the inhibitory effect of alcohol on their cariogenic flora. Alcohol may also enhance the release of fluoride from certain restorative materials. This, and the possibility that they probably sought and received dental care less frequently, may explain why they have fewer filled teeth. Unfortunately, we did not have data on the frequency of their dental visits. 

 Our multivariate analysis that took into account several potential confounders confirmed that the “alcohol and drug” abuse group had a 38% higher risk of having decayed teeth compared to the “alcohol only” group. Our attempt to see if beer drinking alone (which contains higher levels of fluoride) would explain this lower risk for decayed teeth among “alcohol only” group failed to yield statistical significance but was in the anticipated direction (OR = 0.92; *P* = .66). The fact that the units of alcohol consumed per week within each group did not make a significant difference in the risk for decayed teeth (OR = 1.001; 95% CI = 1.0–1.002), perhaps indirectly supports that it is the beer drinking that reduces the risk for the D component of caries.

 Finally, when we explored the risk factors for the higher F component of caries while controlling for the known confounders, we saw that males who belonged to a higher social class and drank wine were the ones who had more filled teeth (Table 3). This is what one would expect. Beer drinkers in this model also had a lower risk of having a higher F component (OR = 0.83) but that association failed to reach statistical significance (*P* = .31). 

 As we have stated, there are limitations in this study. However, it addresses an important scientific question that has not been addressed sufficiently before. Even though both alcohol and drug abuse, either independently or in combination, are deleterious to overall health, understanding the true nature of the effect of these harmful exposures on various components of dental caries experience is worthy of further scientific investigation.

## Supplementary Material

Questionnaire used in the study to gather data on smoking, alcoho and drug use.

## Figures and Tables

**Table 1:** Demographic and behavioural characteristics of the study subjects.

Variable	Alcohol only (*N* = 363)	Alcohol + drug use (*N* = 300)	*P*
Age (y): mean (SD)	43.51 (8.81)	35.41 (7.30)	<.001
Gender (%): M/F			.26
Male	288 (79.3%)	226 (75.3%)	
Female	75 (20.7%)	74 (24.7%)	
Race (%): W/B/A			.15
White	339 (93.4%)	274 (91.3%)	
Black	11 (3.0%)	18 (6.0%)	
Asian	13 (3.6%)	8 (2.7%)	
Duration of abuse (years)	22.93 (10.34)	16.63 (8.54)	<.001
Alcohol units (per week)	286.02 (126.23)	280.91 (119.38)	.60
Current smoking (%)	306 (84.3%)	285 (95.0%)	<.001

**Table 2:** Remaining teeth and caries status by group.

Variable Mean (SD)	Alcohol Only (*N* = 363)	Alcohol + Drug Use (*N* = 300)	*P*
Total Teeth Present	23.24 (6.63)	26.17 (4.59)	<.001
Decayed	0.95 (1.71)	1.31 (2.50)	.032
Missing	8.75 (6.64)	5.81 (4.59)	<.001
Filled	8.09 (5.52)	8.53 (5.30)	.30
DMFT	17.79 (6.87)	15.67 (6.65)	<.001

**Table 3:** Factors associated with decayed teeth and filled teeth (bivariate analyses at 10% level of significance and multivariate logistic regression analysis.

Bivariate analyses^(a)^	OR	95% CI	*P*
Decayed teeth (0 versus >0):			
White versus other	2.38	1.22–4.64	.01
“Alcohol and drugs” abuse versus “alcohol only”	1.34	0.98–1.82	.07
Units of alcohol per week	1.001	1.000–1.003	.04
Filled teeth (0–8 versus >8):			
Male	0.63	0.44–0.91	.013
Higher social class	2.16	1.57–2.98	<.001
Beer drinking versus no beer drinking	0.69	0.49–0.97	.031
Wine drinking versus no wine drinking	2.11	1.34–3.33	<.001
Multivariate analyses^(a)^			
Decayed teeth (0 versus >0):			
White versus other	2.26	1.15–4.45	.018
“Alcohol and drugs” abuse versus “alcohol Only”	1.38	1.01–1.89	.049
Filled teeth (0–8 versus >8):			
Male	1.30	0.89–1.91	.18
Higher social class	1.98	1.43–2.75	<.001
Beer drinking versus no beer drinking	0.83	0.58–1.19	.31
Wine drinking versus no wine drinking	1.85	1.16–2.96	<.05

^(a)^Decayed teeth cut-off median of 0 versus >0 and filled teeth cut-off median of 8 versus >8.
