# Supplementary material for: Tooth Decay in Alcohol Abusers Compared to Alcohol and Drug Abusers
Source: Int J Dent. 2010 Apr 1;2010:786503. doi: 10.1155/2010/786503 (PMC2850152; doi:10.1155/2010/786503)
Supplement: Supplementary file 1 — Questionnaire used in the study to gather data on smoking, alcoho and drug use. [file 786503.f1.pdf]

Appendix:

QUESTIONNAIRE ON SMOKING, ALCOHOL and DRUG USE

Individual number:	

This questionnaire is intended to find out about smoking, alcohol and drug use. It is totally confidential and the information will not be passed on to anyone.

Pl. circle the correct answer for | YES | NO | questions, and fill in other blank boxes: [ ]

SMOKING

1 Have you ever smoked? — YES | NO

Have you smoked in the last year? — YES | NO

If 'YES': How many do you smoke each day on average? — [ ] Type:

How old were you when you started smoking? — [ ] years old

2 Have you given up smoking? — YES | NO

When did you quit? — [ ]

ALCOHOL USE

3 Do you drink alcohol regularly — YES | NO

Have you drunk alcohol in the last year — YES | NO

If 'YES' how many units do you drink of each of these in an average week?

Spirits		units
Beer/ lager		units
Wine		units
Cider		units
Other....................		units

(1 unit is a half pint of beer, a glass of wine or a single measure of spirit)

4 How old were you when you started drinking alcohol? — [ ] years old

5 Have you given up alcohol drinking? — YES | NO

When did you quit? — [ ]

DRUG USE

6 Have you ever used drugs? — YES | NO

Have you used drugs in the last year? — YES | NO

If 'YES' which drugs do you consume or have used before?
(Indicate name, dose, and frequency (i.e., once daily)

7 How old were you when you started using drugs? — [ ] years old

8 Have you given up drug use? — YES | NO

When did you stop using drugs? — [ ]
